# Heads for learning, tails for memory: reward, reinforcement and a role of dopamine in determining behavioral relevance across multiple timescales

**DOI:** 10.3389/fnins.2013.00175

**Published:** 2013-10-11

**Authors:** Mathieu Baudonnat, Anna Huber, Vincent David, Mark E. Walton

**Affiliations:** ^1^Department of Experimental Psychology, University of OxfordOxford, UK; ^2^Department of Psychiatry, University of OxfordOxford, UK; ^3^Centre National de la Recherche Scientifique, Unité Mixte de Recherche 5287, Institut de Neurosciences Cognitives et Intégratives d'Aquitaine, Université de BordeauxTalence, France

**Keywords:** dopamine, nucleus accumbens, hippocampus, reward, reinforcement learning, long term memory

## Abstract

Dopamine has long been tightly associated with aspects of reinforcement learning and motivation in simple situations where there are a limited number of stimuli to guide behavior and constrained range of outcomes. In naturalistic situations, however, there are many potential cues and foraging strategies that could be adopted, and it is critical that animals determine what might be behaviorally relevant in such complex environments. This requires not only detecting discrepancies with what they have recently experienced, but also identifying similarities with past experiences stored in memory. Here, we review what role dopamine might play in determining how and when to learn about the world, and how to develop choice policies appropriate to the situation faced. We discuss evidence that dopamine is shaped by motivation and memory and in turn shapes reward-based memory formation. In particular, we suggest that hippocampal-striatal-dopamine networks may interact to determine how surprising the world is and to either inhibit or promote actions at time of behavioral uncertainty.

## Introduction

It is often assumed, when faced to an unfamiliar environment, that our main task is to learn about this new world via a process of exploration, gathering information through trial and error. As experimenters trying to study these processes, we present our subjects with novel stimuli, different ways of responding and different types of reinforcer in order to determine how they learn about these elements of their world. Sometimes then we might change associations or add new cues to try to understand how new information is represented or existing associations modified. Indeed, when using animals as our subjects, often a lot of effort is expended to ensure that the task environment shares few, if any, features that the animals might have encountered at a previous point in their lives to ensure that new learning can proceed uncontaminated by past experience. Several decades of work has helped to map out how learning and adaptive behavior in these types of environment might be represented in brain circuits, with differing angles of focus within widespread frontal-temporal-striatal networks and particularly their interactions with the neurotransmitter dopamine.

However, while these processes are inarguably critical for survival, this concentration of research into such constrained task set-ups has also indirectly diminished the amount of work on another critical factor in everyday learning and decision making. For what has sometimes been overlooked is the degree to which behavior in novel or changing environments relies not only on detecting discrepancies with what has recently been experienced, but also with identifying similarities with past situations stored in memory (Lisman and Grace, 2005; Gershman and Niv, 2010; Shohamy and Adcock, 2010). While the former process can be used to determine how the environment has changed, the latter in addition can provide a structure, based on past experience, to allow learning to proceed more rapidly. Ideally, these processes will interact dynamically to enable the rapid acquisition of beneficial behaviors in new situations by providing potential response strategies or by biasing attention to what are expected to be the relevant parts of the environment. However, the balance between relying on past strategies or adopting a new response pattern is delicate and in certain circumstances, particularly when the environment has fundamentally changed, a reliance on stored experience at the expense of new learning may also lead to inflexible and maladaptive responses.

A key question, therefore, is what role dopamine transmission might play in guiding *how* to learn and determining when to use or ignore choice policies implemented in previous comparable situations. While dopamine has tended to be tightly associated with aspects of reinforcement learning and motivation, there is increasing evidence that the pattern of dopamine activity can be shaped by both an animal's experience of the structure of an environment and even the long-term nutritional effects of a reinforcer. Moreover, as well as signaling reward predictions, dopamine activity and release can be influenced by novel cues and environments, and therefore might signal the potential importance of elements of the world in order to guide behavior toward the most valuable options (Ljungberg et al., 1992; Kakade and Dayan, 2002; Lisman and Grace, 2005). However, beyond its role in guiding ongoing learning, there is another literature implicating dopamine in aspects of memory consolidation and retrieval at times distant from the original experience (Packard and White, 1989; Floel et al., 2008; Goto and Grace, 2008; Phillips et al., 2008; Shohamy and Adcock, 2010).

In this article, we will attempt to pull together these different strands—learning, familiarity, and memory—to provide a descriptive account of how dopamine transmission might facilitate adaptive behavior in complex, changing environments. Given the heterogeneity of dopamine responses in different terminal regions, for the sake of simplicity, we will focus most closely on phasic dopamine transmission in the ventral striatum/nucleus accumbens (NAc)—in other words, a transient change in dopamine levels lasting between a few hundred milliseconds and several seconds—and how this can have an impact on both short- and longer-term behavior. However, we acknowledge that there are likely different functions of dopamine activity measured across minutes, hours and even days, which might very well correlate with different phases of behavior (see, for example, Schultz, 2007). Moreover, dopamine release in other terminal regions, such as prefrontal cortex, may subserve similar but distinct roles in behavioral flexibility owing to the differences in receptor location and difference in clearance mechanisms and timing (Floresco, 2013).

Here, we will first review the evidence for the modulation of dopamine over different timescales and then will discuss what the behavioral consequences of such modulation would be in terms of patterns of dopamine release in terminal regions. Beyond a straightforward role in reinforcement learning, we will argue that phasic dopamine release here might act as a signal to motivate animals to engage with options at times of uncertainty in order to learn the best predictors of reward in an environment. In a final section, we will outline ideas, building on the work of several other groups (Lisman and Grace, 2005; Johnson et al., 2007; Shohamy and Wagner, 2008; Pennartz et al., 2011), about how hippocampal-striatal-midbrain networks might cooperate to allow such adaptive behaviors to emerge. Specifically, we postulate that this network is key to determine how surprising the world is and therefore how to use memory to shape learning.

## Dopamine across the timescales

### Dopamine, reinforcement, and online reward learning

Reinforcers drive the everyday life of all individuals. Reinforcement can either involve punishment (e.g., pain) and induce avoidance behavior or be positive (e.g., reward) and motivate approach behavior. Unexpected delivery of a reward causes a brief increase in firing rate in a large population of putative midbrain dopamine containing cells as well as a phasic dopamine release in part of the ventral striatum such as the nucleus accumbens (Schultz, 1997; Day et al., 2007; Flagel et al., 2011). A prominent theory suggests that these signals do not directly encode the affective properties of the reward, but instead reflect the deviation at a particular moment in time between an animal's expectation of reward and new information about future rewards (Montague et al., 1996; Schultz, 1997; Aggarwal et al., 2012). This discrepancy—termed a reward prediction error (RPE)—can be used as a teaching signal by temporal difference learning models to enable learning about the long-term cached reward values associated with stimuli in the environment. In support of this idea, dopamine cell activity reflects whether stimuli provide new, useful information about the world (Waelti et al., 2001) and optogenetic driving of the dopamine system to artificially signal the presence of new information when stimuli are presented can cause reward associations to be formed (Steinberg et al., 2013). A similar quantitative and causal relationship has also been demonstrated for dopamine, RPE and action updating in instrumental tasks (Bayer and Glimcher, 2005; Adamantidis et al., 2011). Although fMRI can only provide an indirect measure of dopamine transmission via changes in blood oxygen level-dependent (BOLD) signals (Knutson and Gibbs, 2007), BOLD responses in human ventral tegmental area (VTA) and ventral striatum/NAc have also been shown to represent positive RPEs (D'Ardenne et al., 2008).

While most theoretical work has focused on this type of reward-driven dopamine activity, it is increasingly clear that there is a heterogeneity of response types that can be observed in rodents and primates between different putative dopamine neurons. For instance, while some dopamine containing neurons are modulated by the expected value of predictive stimuli, showing an increase in firing that scales both with anticipated future reward *and* a decrease in firing that scales with anticipate future punishment (e.g., Matsumoto and Hikosaka, 2009; Cohen et al., 2012), other neurons appear mainly to reflect anticipated future reward alone (Mirenowicz and Schultz, 1996; Joshua et al., 2008). Yet another population scales with the likelihood of any future relevant event, whether positive or negative (Matsumoto and Hikosaka, 2009), which has been suggested to be a signal encoding the motivational salience of a stimulus (Bromberg-Martin et al., 2010a). This latter response may relate to the well-known observation that dopamine neurons can briefly respond to unexpected novel stimuli, which have no direct association with any reinforcer (Ljungberg et al., 1992; Horvitz et al., 1997). Alternatively, these responses to novel stimuli could reflect a signal to promote exploration to gain new information (Kakade and Dayan, 2002; Bromberg-Martin and Hikosaka, 2009). A similar range of responses to rewards and punishment can also be seen at the time of reinforcer delivery (Brischoux et al., 2009; Matsumoto and Hikosaka, 2009; Fiorillo et al., 2013a).

Moreover, recent studies have shown that the unfolding activity patterns of an individual dopamine neuron may also encode multiple signals across different timescales. Some dopamine neurons have been shown to exhibit a short-latency, brief phasic response that correlates with reward value, followed by a slower change in activity that scales with reward uncertainty (Fiorillo et al., 2003). In other studies, dopamine cell firing may initially code for the initial surprise and/or intensity of a stimulus before evolving to signal the motivational value of an upcoming outcome (Nomoto et al., 2010; Fiorillo et al., 2013b). The same population of dopamine neurons may also come to reflect different parameters of the local reward environment at different points in a trial (Bromberg-Martin et al., 2010c).

In spite of the fact that dopamine transmission has often been viewed as a regionally−homogenous reinforcement signal broadcast to all terminal regions (Schultz, 2002), a range of dopamine release patterns has been observed in different striatal sub-regions. For instance, changes in extracellular dopamine levels measured with microdialysis in response to cues predicting either reward or punishment, or to the receipt of reward or punishment itself, can be different in the NAc core or shell regions (Ito et al., 2000; Pezze et al., 2001; Bassareo et al., 2002). The same is also true for brief phasic changes in dopamine measured at sub-second time resolution with fast-scan cyclic voltammetry, with variations observed across different parts of the NAc (core vs. shell) and dorsal striatum (Brown et al., 2011; Badrinarayan et al., 2012). Whether these differences relate to the anatomical diversity that was described above (Brischoux et al., 2009; Lammel et al., 2011), patterns of afferent input to the dopamine cells in the midbrain (Besson et al., 2012; Tolu et al., 2013), local influences on dopamine release from afferent input in the terminal region (Floresco et al., 1998; Threlfell et al., 2012), differences in the temporal resolution of electrophysiology compared to electrochemistry and microdialysis (Schultz, 2007), or combinations of all is an area of active research.

Taken together, all the above evidence demonstrates that the dopamine system is able to reactively signal events of potential significance, to reflect the discrepancy between current predictions and the discounted sum of prospective rewards, and to update these predictions as new information is acquired, which are prerequisites of trial-and-error, model-free, associative learning. Even though the precise degree of heterogeneity of dopamine cell responses remains contentious (Fiorillo et al., 2013b; Schultz, 2013), there are clearly diverse dopamine release patterns across striatum. As we will go on to discuss, there is also increasing evidence that the dopamine systems may not simply encode such short-term information, but may as well interact with other structures to allow stored information about task structure and motivational parameters to influence dopamine release. This, we will argue, may enable dopamine to provide a signal that influences what to learn and how to behave in particular contexts.

### Dopamine responses sculpted by memory and motivation

While it is well known that, in agreement with RPE theories, dopamine cell activity and dopamine release in the NAc adapts during associative learning to reflect the earliest consistent predictor of future reward and experienced history of reinforcement (Schultz, 1997; Nakahara et al., 2004; Flagel et al., 2011), it is also apparent that dopamine in response to cues and outcomes can be shaped by task structure and memory. For instance, dopamine cell activity at the time of reward delivery scales to the potential range of available outcomes signaled by a stimulus, with a similar increase in activity across a 10-fold range of reward sizes (Tobler et al., 2005). Moreover, after extensive training on tasks where the consequences of one trial have direct impact on the likelihood of reward in a subsequent trial (e.g., a deterministic reversal learning task where one option is always rewarded and the other not or a sequential response task where one target is always rewarded within a block of 4 trials), some dopamine neurons come to represent values partially inferred from the overall reward structure rather than just from direct recent experience (Nakahara et al., 2004; Bromberg-Martin et al., 2010b). In human fMRI studies, ventral striatal/NAc BOLD signals have been shown to incorporate information about task structure in the RPE signals (Daw et al., 2011) and, intriguingly, it has recently been shown that systemic L-DOPA enhances the use of such model-based information (Wunderlich et al., 2012).

Studies of dopamine release in the striatum have also found that training can selectively modulate transmission in different regions. As discussed in an earlier section, unpredicted reward given to naïve animals only caused increases in dopamine in the NAc core region and not in the NAc shell, or the dorsomedial or dorsolateral striatum. However, after training on a simple cued instrumental task, now unpredicted reward *did* evoke dopamine release in the dorsomedial striatum as well as the NAc core (Brown et al., 2011). While this might just reflect an increase in the number of dopamine neurons recruited following any reinforced training, the fact that this occurred outside the task structure suggests this change could instead relate to the fact that reward has become a relevant event for guiding responding.

**Figure 1:**
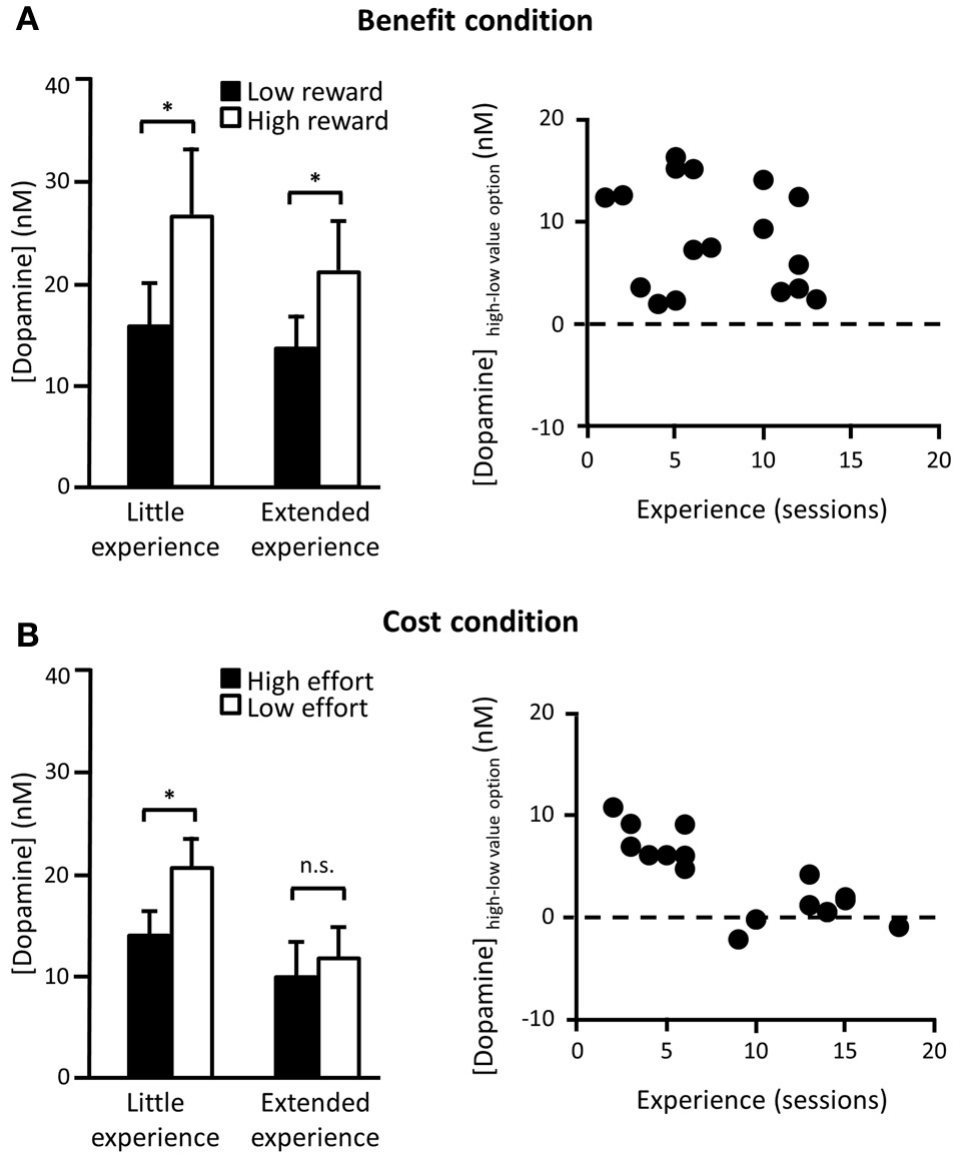
**Cue-elicited dopamine release in NAc core during a two-option decision making task. (A)** Left panel: Average (mean + s.e.m.) phasic dopamine release in NAc core elicited by cues signaling availability of either a known low (2 pellets) or a high reward (4 pellets) option for a fixed cost (16 lever presses) in rats who have either had little (<9 sessions) or extended (≥9 sessions) experience with these contingencies. Data taken from forced trials (where only one option was available) after animals were choosing the high reward option on ≥75% of choice trials. Contingencies changed every session so rats had to flexibly update associations in each session. Right panel: Difference in cue-evoked peak dopamine release between cues signaling high and low reward options as a function of number of sessions in which they had experienced these contingencies. There was no correlation between experience of dopamine-based benefit encoding. **(B)** Left panel: Same as **(A)** except now the benefit was fixed (2 pellets) and the cost varied across options (low effort = 2 lever presses; high effort = 16 lever presses). Right panel: Same as **(A)** for high and low effort costs. Now there was a significant reduction in dopamine cost encoding as a function of experience. *n.s*. differences not significant; ^*^significant at *p* < 0.05. [adapted from Gan et al. (2010)].

Decision parameters that drive dopamine transmission can also be dissociably influenced by the amount of experience of the task. In one recent study, rats were trained on a two-option decision making paradigm, where one option required a particular amount of work to gain a particular size of reward and either the work or reward of the alternative was systematically varied. This meant that animals' choices were either guided by a difference in the effort required to gain the reward (different cost, same benefit) or by a difference in the eventual payoff for taking a particular option (same cost, different benefit). In the “benefit” conditions, dopamine release elicited when either of the two options was presented consistently reflected the anticipated future reward associated with that option. This cue-elicited effect was unchanged over a range of testing sessions (Figure 1A). However, a different pattern was observed in the “cost” conditions. Now, differential dopamine release to the low as compared to the standard cost option was only recorded in early training, when the change in effort was unexpected, but after several sessions of experience this difference disappeared (Gan et al., 2010) (Figure 1B). This was not caused by any detectable differences in behavior as the animals showed equal preference for the low effort and the high reward options and continued to rapidly update their responses when the cost-benefit contingencies changed across sessions.

Exactly why and when dopamine adapts in response to the task structure and what role dopamine plays in shaping learning of task structure is currently a matter of some debate (Gershman and Niv, 2010; McDannald et al., 2012; Nakahara and Hikosaka, 2012). In the cost-benefit study described above, one idea is that NAc dopamine release is reflecting uncertainty over the temporal statistics of reward delivery. In the cost conditions, even though the cue-to-reward period varies according to the animals' choice, the overall average trial-to-trial reward rate is relatively static as the inter-trial intervals (ITIs) were adjusted as a function of the animals' choices. Such a proposition is supported by recent evidence from a Pavlovian task, which showed that NAc dopamine adapts to the temporal variability of cue-reward pairings over training. More specifically, cue-elicited dopamine decreased after extensive presentation of the cue-reward associations, although was then restored if the cue was unexpectedly presented at a shorter ITI (Clark et al., 2013). A second, related idea is that, while dopamine may preferentially encode the anticipated benefits of a course of action, there may also be an initial boost in dopamine release to any unpredicted, uncertain event to motivate exploration and investigation of that option (Kakade and Dayan, 2002; Phillips et al., 2007; Walton et al., 2011). Once a settled pattern of responding has been established in a stable environment, however, the NAc dopamine signal may not be required to sustain performance.

All of the above studies have looked at how dopamine is shaped by learned or inferred predictions of proximal rewards. However, it is important to remember that dopamine transmission is also strongly affected by motivational state. While food reward consistently increases dopamine efflux in hungry animals, the effect is much reduced in sated rats (Wilson et al., 1995; Bassareo and Di Chiara, 1999). This effect appears specific to sensory properties of a particular food as while there is no increase in dopamine levels in the NAc measured with microdialysis when presented a food type that had previously been consumed, dopamine efflux in this situation still occurs when given a novel foodstuff (Ahn and Phillips, 1999). Such effects may reflect an important modulatory role of peptides such as insulin, leptin, and ghrelin, which act on neurons in the VTA and regions that target the VTA such as the lateral hypothalamus and can therefore affect dopamine transmission (Abizaid, 2009; Domingos et al., 2011; Mebel et al., 2012).

Dopamine transmission is also sensitive to distal post-ingestive effects, such as the nutritional, calorific and metabolic consequences of particular rewards (de Araujo et al., 2008). Therefore, while unexpected receipt, or cues signaling the impending delivery, of sucrose pellets consistently evokes dopamine release, this is attenuated when a sweet but calorie-free saccharine reward is used instead (Beeler et al., 2012; McCutcheon et al., 2012). Again, it is likely that these effects may be heterogeneous across the striatum depending on task and on the nutritional content. In recent studies looking at the effects of direct, intra-gastric infusions of fat (i.e., bypassing the taste receptors entirely), dopamine levels increased in dorsal striatum whereas NAc core dopamine decreased as a function of fat density (Ferreira et al., 2012). However, it remains to be determined whether dopamine levels update to reflect an inference about how behavior should be prioritized given an animal's current motivational state or instead are only altered after experiencing a particular food in a particular motivational state.

These motivational influences on dopamine are intriguing as they demonstrate that dopamine transmission can be modulated by distal, as well as proximal, consequences of reinforcers, which poses an extreme credit assignment problem given the time that must elapse between the predictive cues, ingestion and the metabolic effects of these rewards. More importantly, they also act as a reminder that theories of dopamine-mediated reinforcement learning and behavior should incorporate motivational parameters. An interesting possibility is that such a mechanism could underpin the seemingly paradoxical decisions that have been observed in the foraging literature where animals' choices depend on memory for context-dependent utility (Pompilio and Kacelnik, 2010).

### Dopamine, synaptic plasticity, and long-term memory

In the previous section, we detailed evidence that dopamine transmission is influenced by memory of reward structure and motivational context. However, it is also important to remember that dopamine has also been implicated directly in long-term memory processes themselves. Long-term potentiation (LTP) and long-term depression (LTD) are thought to be critical at the cellular level to underlie memory formation and long-lasting changes in synaptic plasticity (Kandel, 2001). Dopamine has been identified as a strong modulator of these cellular adaptations (Wickens, 2009; Lovinger, 2010). For instance, D1 antagonists block the induction of LTP in striatum (Kerr and Wickens, 2001) and both D1 and D2 receptors appear necessary for striatal LTD (Calabresi et al., 1992). Dopamine is also required to enable spike-timing dependent LTP or LTD in dorsal striatum (Pawlak and Kerr, 2008). Indeed, it is likely that the precise timing of dopamine transmission at striatal synapses has a significant effect on the direction of plasticity (Wickens, 2009).

At the molecular level, strengthening synapse communication is critical in setting up a network supporting both the acquisition and the recall of a particular learning experience. This relies on the co-release of glutamate and dopamine at target synapses. D1-like and D2-like receptor activation leads to the activation of two competing molecular pathways. While D1 receptors are coupled with a Gαs protein which positively modulates adenylate cyclases, D2 receptors are coupled with a Gαi protein which inhibits adenylate cyclases (Siegel et al., 1999; Hyman et al., 2006). Adenylate cyclases are responsible for the activation of various protein kinases and molecular cascades, which lead to activation of transcription factors (e.g., the phosphorylated form of the cAMP response element binding protein, pCREB) which in turn induce the transcription of immediate early genes (e.g., *c-fos*). The resulting proteins underlie the systemic consolidation necessary for long-term memory storage and recall (Huang and Kandel, 1995; Frankland and Bontempi, 2005).

Much of the work looking at the role of dopamine in memory formation, consolidation and recall at a behavioral level has concentrated on the effects of direct hippocampal dopamine interference (Bethus et al., 2010), which largely goes beyond the scope of this review. However, there are some findings that also imply a role for striatal dopamine itself in the encoding and consolidation of memories. It has been known for a while that dopamine-dependent potentiation of corticostriatal synaptic efficacy correlates with the speed of acquisition of intracranial self-stimulation, which in essence provides a cellular correlate of the standard, short-term reinforcement learning described in earlier sections (Reynolds et al., 2001). However, there are also some more unexpected reported effects of post-training dopamine manipulations on reinforcement that occur long after reward receipt. For instance, Dalley and colleagues reported that infusion of either a D1 or NMDA antagonist into the NAc given *after* a Pavlovian conditioning experiment blocked acquisition of autoshaping responses (Dalley et al., 2005). Similarly, in an inhibitory avoidance task, post-training injection of dopamine in the NAc shell, but not the NAc core, enhanced the retention of the conditioning (LaLumiere et al., 2005).

These studies indicate that dopamine's reinforcing effect can be temporally dissociated from the receipt of reward. Moreover, dopamine may even play some role in consolidation of memories for unreinforced items, similar to the way in which dopamine is activated in response to the presentation of novel stimuli. Dopamine lesions to the NAc core, but not to the shell, impaired a familiarity discrimination test with objects 24 h after the initial presentation, and NAc shell (and core to some extent) dopamine lesions affected location familiarity responses (Nelson et al., 2010).

### Interim summary: dopamine across the timescales

In the above sections, we have described how dopamine acts at both short- and long-term timescales. Its most well defined function is that it allows the detection of discrepancies between predictions and outcomes at the time of an event. At a cellular level, dopamine also plays an important role in the storage of past experiences into memory. However, it is also becoming increasingly clear that all of these effects can be shaped by memory, motivation and internal state. Dopamine cannot be described as providing a homogeneous reinforcement signal as dopamine's role in these processes are clearly both site- and task-specific, with effects in a particular striatal region dependent on the state of the environment and of the animal.

In the next sections, we will build on these points to outline a possible framework that might help explain how phasic dopamine can function at different timescales. In particular, we will focus on two main aspects: (1) the behavioral consequences of dopamine release in striatal regions, and (2) the function of the anatomical networks in which these striatal regions are embedded and how dopamine might facilitate selection of one system over another.

## Dopamine transmission, learning, and strategy selection

### Dopamine transmission across the striatum

A critical issue when considering the role of dopamine concerns the question of what behavioral effect heterogeneous dopamine transmission has in different terminal regions. As has been discussed above, dopamine cell activity in many circumstances correlates highly with RPE signals. However, dopamine *release* in terminal regions in these situations suggests a role beyond a passive process of learning.

Across a range of studies, dopamine in the NAc, particularly in response to cues—and particularly in the core region—is required to activate animals to engage in a behavioral response. For instance, dopamine is only required to learn about cue-reward relationships in situations where cues acquire Pavlovian incentive values, which thus promote approach behavior, rather than simply being predictors of reward (Di Ciano et al., 2001; Dalley et al., 2002; Flagel et al., 2011). Similarly, dopamine transmission in cued decision making tasks seems strongly tied to the advantageous response elicited by a cue, whether to gain reward or avoid punishment, rather than just the predictive cue itself, and in some situations, can be elicited by an internal drive to respond in the absence of any external stimulus (Roitman et al., 2004; Yun et al., 2004; Oleson et al., 2012; Wassum et al., 2012). At least for the NAc core, this may be in the form of a general motivational drive rather than a representation of the particular sensory properties of the outcome. Lesioning the NAc core or blocking D1 receptors in this region disrupts general motivational arousal associated with cues during Pavlovian-instrumental transfer, but the former manipulation has no effect on outcome-specific versions of this task (Lex and Hauber, 2008; Corbit and Balleine, 2011).

In both Pavlovian and cued instrumental situations, dopamine may only be critical when there is some uncertainty or novelty about the environment, whether in terms of the consequences associated with a choice or the particular actions required to obtain a reward (cf. Nicola, 2010). This is not to say that NAc dopamine plays any direct role at the time of a choice in guiding the selection of one alternative over another. Several different studies have shown now that phasic dopamine reflects the anticipated future benefit of whatever option will be chosen, even in cases where this is not the most valuable available option (Morris et al., 2006; Walton et al., 2011, but see Roesch et al., 2007).

Instead, phasic release in NAc may play two complementary roles: first, to act to energize animals to engage with a response based on its anticipated cached value, especially in situations when the environment changes and reward associations need to be updated; and, second, to enable them to learn about *behaviorally-relevant* consequences associated with cues in the environment (Phillips et al., 2007; Nicola, 2010; Walton et al., 2011). Although NAc dopamine is required to promote reward seeking even in the most simple Pavlovian situations, little is currently known about how this occurs in more complex, naturalistic environments where there are multiple potential cues and an unknown range of potential outcomes and it is necessary to determine which parameters are useful to guide behavior. Although we know of no studies to date that have directly investigated this issue with respect to NAc dopamine signaling, there is some recent evidence from fMRI that the ventral striatum/NAc might play an particular role in extracting information relevant for learning about reward (Klein-Flugge et al., 2011). In this study, participants underwent Pavlovian conditioning using stimuli that varied trial-by-trial in both their associated reward magnitude and the delay-to-reward. Interspersed were instrumental timing estimation trials where they had to predict when (but not what size) reward would appear in order to accumulate points that determined how much money they would receive for participating. While both precise reward magnitude and timing prediction errors were observable in the midbrain, as predicted by temporal difference learning models, ventral striatum/NAc BOLD signals *only* reflected the timing RPE signals required to guide subsequent choices and not the task-irrelevant reward RPE signals. Future studies will be needed to determine if these BOLD signal changes are driven by dopamine transmission. However, it may be that NAc core dopamine only signals a subset of relevant events, playing a particular role in motivating animals to learn strategies to improve their current state. As we will discuss in a later section, phasic NAc dopamine does not seem to be required when simply switching behavior to maintain a previous state (i.e., in most reversal tasks) (Haluk and Floresco, 2009).

Inspite of there being a number of examples showing that the patterns of rapid dopamine release are frequently divergent in the NAc core and shell, the function of dopamine in the latter structure is not yet clear. There is some evidence for a potential role of NAc shell in spatial processing, with infusions of a dopamine antagonist decreasing place conditioning but not cue conditioning (Ito and Hayen, 2011) and with dopamine efflux here being influenced by projections from ventral hippocampus (Legault et al., 2000). However, this seems unlikely to define its primary function given that there are many events distinct from spatial context that elicit NAc shell dopamine transmission and, in fact, the ventral hippocampus is arguably also more concerned with emotional responses to uncertainty, conflict detection and response inhibition than with spatial processing (Bannerman et al., 2004; Abela et al., 2013). Instead, it seems possible that NAc shell dopamine is important for signaling the occurrence of novel and potentially salient events, particularly in the case when there is ambiguity over the cause of that event. Tuning down NAc shell dopamine when uncertainty is resolved might facilitate an appropriate allocation of attention to the environment only to behaviorally relevant events. In partial support of this idea, it is notable that, while the NAc core (and NAc core dopamine), but not the NAc shell, has been implicated as being critical for beneficial choice behavior where there is guaranteed reward for any response (for example, effort- or delay-based decision making), the shell region appears to play a more critical role than the core region in biasing decisions when there is uncertainty about reward (Sokolowski and Salamone, 1998; Ghods-Sharifi and Floresco, 2010; Stopper and Floresco, 2011). Intriguingly, there is some evidence suggesting that NAc core and shell dopamine might play complementary and possibly antagonistic functions in some circumstances (Ito and Hayen, 2011), which may reflect the degree to which the overall statistics of the environment are known and how responses are being allocated.

Compared to the mesolimbic pathways to NAc, the nigrostriatal dopamine projections to dorsal striatum have been tied more closely to action selection and action reinforcement. Nonetheless, there is likely no simple, neat divide between the motivational and motor components of dopamine-dependent behavior, not least as the activity of many putative dopamine cells in both substantia nigra pars compacta and VTA correlates with RPE signals (Everitt and Robbins, 2005; Wise, 2009). It is known that dopamine in dorsomedial striatum is necessary to detect the contingency between actions and their consequences (Lex and Hauber, 2010) and, like in the NAc, dopamine levels also track the acquisition of a reinforced instrumental action and are sensitive to satiety (Ostlund et al., 2011). However, compared to NAc, where DA release appears to energize a decision policy selected elsewhere, there is also some evidence that DA in dorsal striatum may directly bias the choices to be made, particularly when there is evidence of a requirement to change behavior. Selective stimulation of the D1- or D2-receptor expressing striatal neurons during a probabilistic decision making task in this region can increase the incidence of a contralateral or ipsilateral action respectively following an unrewarded action (Tai et al., 2012).

Therefore, while there may be a common role of dopamine release across the striatum in helping reduce ambiguity through motivating cue-driven behavior or detecting the consequences of a novel event or response (Costa, 2007; Redgrave et al., 2008), the effect of dopamine transmission will be shaped by the properties of the terminal region and the networks in which this region is embedded. To explore this further, in the next sections, we will consider some examples of how and why this might occur, with a particular focus on NAc dopamine.

### Dopamine, strategy selection, and behavioral relevance in complex changeable environments

In naturalistic situations, the environment consists of multiple cues and there are multiple potential responses that could be made at any one time, the relevance of which change constantly over time. Consequently, a foraging animal will need to rely on different cues to locate food depending on its availability at a particular time, its current motivational requirements and the environment in which the animal is operating, and to update its search strategy accordingly. These various cues and their relations to outcomes can be learned through experience and then retrieved from memory to guide behavior efficiently when the animal is again faced with comparable situations in the future. As we discussed in the previous sections, as well as being important for learning cue- and response-outcome associations through trial-and-error, dopamine transmission can be sculpted by experience and therefore may be critical to guide when and how stored memories are used and updated when the environment changes. To enable appropriate learning decision and decision making to take place, general rules can be established to facilitate trial-and-error learning to which exceptions are then added. We would contend that dopamine is involved in these learning and memory processes by playing a key role in guiding the initial search strategy when environmental contingencies are uncertain, putting the neural network in a state to learn about the consequences of choices, and also in determining when to reactivate parts of this network when encountering novel situations for which previously acquired strategies may be useful.

One prominent brain structure candidate for supporting the switch between updating current estimates of the world, using a previously stored memory or starting new learning is the NAc. Haluk and Floresco (2009) have argued that dopamine in the NAc is not required simply to update behavior when contingencies reverse, but is instead key to allow the shifting from a cue-driven response strategy, where spatial location is irrelevant, to a spatially-guided response strategy, where the cue location now should be ignored. They showed that either pharmacological blockade of NAc D1 receptors or stimulation of D2 receptors impairs this type of strategy shift. Interestingly, the D1 receptor antagonist had no comparable effect when the response strategy was simply reversed, demonstrating that there is not some general role of NAc phasic dopamine in altering choice strategies when there is no change to the overall reward statistics of the environment. In a separate study, it was demonstrated that NAc tonic dopamine levels also markedly increase when rats switch between response strategies, much more so than when initially acquiring the task, as well as in control conditions where the reward contingencies are deliberately kept uncertain (Stefani and Moghaddam, 2006).

Although Haluk and Floresco describe one of their conditions as requiring a “spatial response strategy,” the navigational component in an operant chamber is necessarily sparse and in other settings, such as a water maze (McDonald and White, 1994; Porte et al., 2008), the radial arm maze or a Y-maze, an egocentric response strategy can be dissociable from an allocentric spatial one. In many naturalistic situations, spatial-, response-, and cue-learning will not necessarily proceed independently and the predictive value of each will have to be weighed up against one another, with the optimal choice strategy obviously dependent on the particular task environment. As well as competing for control, these systems may also cooperate during the initial stages of learning (White and McDonald, 2002). Several lines of evidence suggest that partially separate frontal-striatal-temporal networks underpin these different forms of learning and also guide which should be used to guide behavior (White and McDonald, 2002; Porte et al., 2008). Therefore, a key open question in the framework of the current review is to try to pinpoint how dopamine in these interconnected networks may guide attention to the appropriate parts of the environment in order to make advantageous foraging choices.

Other than Haluk and Floresco (2009), few studies to date have directly tried to address how dopamine might help arbitrate between spatial- and cue-guided behavior. The first clue came from the work of Packard and White (1991) in which they manipulated dopamine after training their animals in either a “win-shift” or “win-stay” task, which they had previously shown to depend on the hippocampus or caudate nucleus, respectively. They found that immediate post-training injection of dopamine agonists in the dorsal hippocampus selectively improved win-shift retention whereas injections into the posterior ventrolateral caudate nucleus improved the acquisition of the win-stay task. They argued that dopamine acts to modulate the functioning of the structure into which it is infused and potentially acts to reduce the interference of one strategy over another in the early stage of learning that can slow down the acquisition of the task (Packard and White, 1991).

**Figure 2:**
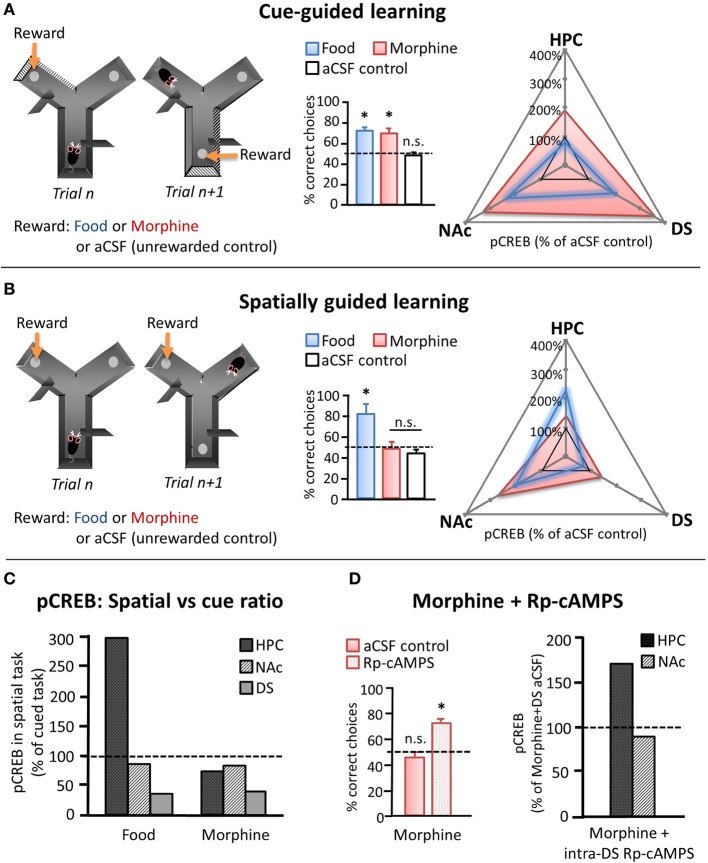
**Effects of natural and pharmacological reinforcers on the acquisition of cue- and spatially-guided learning strategies. (A, B)** Left panels depict schematics of a cue-guided **(A)** and a spatially guided **(B)** version of a Y-maze decision making task. Correct responses were guided either by intramaze visual cues or by spatial location, respectively, and were reinforced in separate groups of mice by either food reward or intra-VTA morphine infusions. Control mice received intra-VTA aCSF infusions and no food reward at the “correct” location. Middle panels depict choice performance on the 10th day of training on the respective task (chance performance = 50%, marked with dashed line). Right panels depict pCREB levels measured in NAc, dorsal striatum (DS), and the hippocampus (HPC) of food- and morphine-reinforced mice after 10 days of training on the cued (**A**: upper) or the spatial (**B**: middle) version of the Y-maze task, normalized to pCREB levels observed in aCSF controls. **(C)** Relative changes in pCREB levels in HPC, NAc, and DS after training on the spatial as compared to the cued task (cued task pCREB = 100%, dashed line). **(D)** Left: Effect of daily intra-DS injection of either the PKA inhibitor Rp-cAMPS or aCSF on the spatial version of the Y maze in morphine reinforced mice. Left: Choice performance on the 10th training day. Right: pCREB levels measured in NAc and HPC after 10 days of training on the spatial task in morphine-reinforced mice that received intra-DS injections of Rp-cAMPS, normalized to morphine-reinforced aCSF controls (dashed line). *n.s*. differences not significant, ^*^significant at *p* < 0.05. [adapted from Baudonnat et al. (2011)].

Recently, Baudonnat et al. (2011) also investigated how different types of reward influence the selection and acquisition of learning strategies. Initially, they showed that mice were able to learn whether to use spatial location or intramaze visual cues to guide decision-making in a Y-maze when correct choices were reinforced with natural (food) reward (Figures 2A,B). As discussed in a previous section, it is well known that unpredicted reward drives phasic dopamine and that dopamine transmission at target synapses can induce the activation of molecular pathways leading to CREB phosphorylation and modification of synaptic strength (Dudman et al., 2003). Therefore, in order to investigate the cellular mechanism involved in the different types of learning, they measured pCREB levels in different candidate brain regions after the last behavioral testing session. Similar to the study by Packard and White (1991), pCREB was found to be specifically increased in the hippocampus after acquiring the spatial task whereas the increase was mainly present in the dorsal striatum after the cued task. By contrast, pCREB was highly expressed in the NAc independently of the task type (Figure 2C).

The above example demonstrates the cellular effects in hippocampus—dorsal striatum—NAc regions during appropriate reinforcement learning. However, drugs of abuse can also pharmacologically hijack the dopamine system and result in maladaptive patterns of behavior. To determine how an excess of dopamine might affect learning strategies, Baudonnat and colleagues carried out the same Y-maze experiment except that instead of receiving food reward for correct responses, the mice received intra-VTA injections of morphine, which has been shown to disinhibit dopamine neurons and induce dopamine release in target structures (Matthews and German, 1984; Johnson and North, 1992; Nugent et al., 2007; Baudonnat et al., 2011). While the animals learned the cued version of the task at a comparable rate with either natural or pharmacological reward, mice reinforced with morphine were unable to acquire the spatial strategy (Figure 2B). pCREB staining demonstrated that morphine given for correct responses caused increased dopamine-related plasticity in both the NAc and the dorsal striatum in both tasks but was correlated with a marked decrease in pCREB expression in dorsal hippocampus in the spatial task compared to when reinforced with food.

One possibility was that the increase in dorsal and ventral striatal dopamine release originating from the intra-VTA morphine injection, even to predicted rewards, would bias choices to be driven by striatal networks at the expense of hippocampal ones. In support of this hypothesis, inhibition of the protein kinase A pathway in the dorsal striatum, thus potentially down-regulating the consequences of drug-induced dopamine transmission, enabled mice to learn the spatial task even with intra-VTA morphine as the reward and restored pCREB expression in the hippocampus (Figure 2D).

This study leaves open several interesting questions concerning how exactly dopamine transmission might dynamically regulate how learning and attention are flexibly allocated and what the longer-term behavioral consequences of this type of dopamine-driven synaptic plasticity might be when there are multiple potential strategies to guide foraging choices. Nonetheless, it clearly demonstrates that different reinforcers, and by extension dopamine transmission, can have a strong influence on the acquisition and consolidation of an appropriate choice strategy, and which brain regions/systems are prioritized during decision making. Similarly, it also shows that the parameters of normal reward-guided dopamine release and the resulting dopamine-mediated synaptic plasticity are critical to allow the correct information to be identified and retained. Thus, modulation of the magnitude and timing of dopamine release may be key to allowing animals to determine when to use, update or discard past experience when encountering novel situations.

### Temporal lobe influences on dopamine transmission during learning and strategy selection

In the previous sections, we have tried to highlight the fact that short- and more long-term dopamine-dependent processes are not mutually exclusive but instead are mutually interacting in order to produce adaptive behavior. Moreover, dopamine transmission in target structures can be locally modulated and may therefore act locally to facilitate acquisition and selection of appropriate foraging strategies. What is not yet clear is: (1) how dopamine transmission affects the way in which potentially competing networks for valuation and behavior are selected; and (2) how dopamine release in terminal regions and afferent modulators of dopamine transmission interact to signal the current state of the world. In the remainder of this review, we will briefly outline some ideas about these processes. Specifically, we will focus on how dopamine transmission may interact with hippocampal circuits through the midbrain and NAc in a bi-directional manner to allow us to learn and behave appropriately in uncertain and changing environments.

As briefly discussed in a previous section, there are strong inputs from the hippocampus into the NAc in both rats and primates (Brog et al., 1993; French and Totterdell, 2002; Friedman et al., 2002), particularly—though not exclusively—from ventral hippocampus to the medial NAc in primates/NAc shell in rodents (NB. the core and shell are difficult to characterize based on cytoarchitecture in primates). In rodents, some of these synapses may converge with amygdala and medial frontal cortex inputs (French and Totterdell, 2002, 2003) and may predominantly target NAc medium spiny neurons associated with the direct pathway (MacAskill et al., 2012). These circuits are also involved in the regulation of VTA DA neuron firing and excitability (Floresco et al., 2001; Lodge and Grace, 2006). Grace et al. (2007), in particular, have highlighted the potential importance of the hippocampus—NAc—ventral pallidum—VTA circuit for altering the activity states of midbrain dopamine neurons, which can therefore act to gate glutamate-driven burst firing. By extension, the modification of the basal activity of DA neurons makes them more likely to produce phasic burst firing when a novel event is detected or when a reward-predictive cue is presented (Grace et al., 2007; Aggarwal et al., 2012). However, this loop is not the only one involved in regulating VTA DA neuronal activity. Several hippocampal—VTA networks, coexisting via different relays in the brain (such as the lateral septum), modulate dopamine cell activity, along with multiple other pathways from cortical regions such as the orbitofrontal and medial frontal cortex (Lodge, 2011; Luo et al., 2011). The precise contribution of the inputs to midbrain dopamine cells or the afferents to striatal regions targeted by dopamine fibers to patterns of local dopamine *release* remains to be determined.

How might these circuits facilitate learning and adaptive foraging through NAc dopamine? As we described in the previous sections, phasic dopamine release in the NAc appears not only to correlate with predictions of future rewards and deviations from these predictions, but can also be driven by novel stimuli, shaped by uncertainty about the structure of the environment and about what information should be used to guide choices, and modified by motivational state and the post-ingestive consequences of reward. At a behavioral level, dopamine release, particularly in the NAc core, can promote approach behavior to cues in the environment, though is unlikely to play a leading role in setting a behavioral policy. Moreover, though dopamine release in NAc in response to both reward-predicting cues and unexpected reward may often be formally consistent with an RPE (i.e., both signal a deviation from a past prediction of future rewards based on newly received information), the two signals may in fact be regulated via dissociable processes (Wanat et al., 2013). This potentially allows for separable influences of afferent structures on cue- and outcome-driven dopamine transmission.

While the main function of the hippocampus is often described in terms of spatial memory, there is also an extensive literature demonstrating that this brain structure also plays a key role in encoding the predictability and regularity of events as well as signaling mismatches or conflicts in the incoming information (Honey et al., 1998; Gray and McNaughton, 2000; Strange et al., 2005; Kumaran and Maguire, 2007; Sanderson and Bannerman, 2012; Schapiro et al., 2012). Given the anatomical and functional connections between the hippocampus, dopaminergic midbrain and the NAc, there is the possibility that these circuits interact dynamically not only to detect novelty, but also to determine the *behavioral relevance* of a new ambiguous cue or environment. One way this might occur is through hippocampal modulations of outcome-driven dopamine release (how surprising is a cue given the current state of the environment and how unexpected is the outcome given past expectations in this state), which in turn can influence synaptic plasticity and the efficiency with which particular inputs to the NAc can affect activity in this region (see Floresco, 2007). This may well be a bi-directional mechanism, with deviations in stimulus-surprise (associative or contextual novelty) being directed from hippocampus to influence mesolimbic dopamine release and the magnitude of outcome-surprise signaled by the extent of NAc dopamine release, which is then directly or indirectly communicated to temporal lobe structures. For instance, as suggested by Gershman, Niv and colleagues in a series of papers (Gershman et al., 2010; Gershman and Niv, 2010), the magnitude of an RPE signaled by dopamine release might be used to indicate whether or not an environment has fundamentally changed and therefore whether previous choice strategies should be updated, consolidated or discarded in favor of a new state. Therefore, if there is an abrupt change in reward contingencies (for instance, going from a seldom-reinforced to a fully-reinforced situation), the consequent sudden change in outcome-driven dopamine could provide a signal in terminal regions that the animal is in a new context (or new task state, in reinforcement learning terms) and should therefore not integrate the new evidence with past events but should instead treat them as separate situations and start learning anew.

These anatomical loops can therefore potentially account for the effect of dopamine in both short-term encoding of reward associations and longer-term memory recall and updating. The presentation of a familiar situation can cause the re-activation of the memory related to this state. At this time point, the pattern of the cue-outcome contingencies within a behavioral strategy will be critical as to whether the memory is used, updated or discarded because of the labile nature of a re-activated memory (Kuhl et al., 2010). Therefore, by acting in concert, hippocampus – NAc – dopamine circuits could allow organisms not only to work out what cues are relevant in the environment, but also, when faced with a seemingly familiar situation, to determine whether or not to generalize based on stored experience (Shohamy and Wagner, 2008; Wimmer et al., 2012). Specifically, through signaling how similar the current state of the environment is to stored associations—the cues and context via the hippocampus and accompanying reward contingencies via dopamine—these circuits may signal when to integrate separate events if there are statistical regularities between them or, by contrast, when to separate memories and promote new reinforcement learning if there are notable discrepancies.

However, as well as shaping learning and strategy selection, another potential key role of the hippocampus may be the modulation of dopamine-dependent Pavlovian approach behavior by suppressing inappropriate choices or facilitating exploratory responses. Several lines of evidence suggest that one output of hippocampal computations may be to inhibit ongoing behavior in order to prevent impulsive, disadvantageous behavior (Gray and McNaughton, 2000; Mariano et al., 2009; Bannerman et al., 2012; Abela et al., 2013). Therefore, in situations, for instance at times of uncertainty, where there is conflict over which cues in the environment are most relevant to guide behavior or where a superficially tempting option should be resisted in order to obtain a larger benefit in the future, the hippocampus may act as a regulator of NAc dopamine transmission, reducing the likelihood of a prepotent cue-driven response being elicited before the potential future consequences are considered. Consistent with this, Floresco and colleagues found that preventing hippocampal afferents from interacting with NAc dopamine transmission using an asymmetric disconnection procedure caused rats to inappropriately return to previously sampled arms in a foraging task, suggesting that these circuits are required to suppress previously reinforced spatial behaviors (see Floresco, 2007). Conversely, novelty-induced dopamine release to unexpected cues with no current reward associations could provide the motivational drive for animals to approach such a cue in order to gain information about its significance.

In summary, by influencing dopamine transmission in the NAc, the hippocampus can help the dopamine system to: (1) shape appropriate behavior toward the behaviorally- and motivationally-relevant elements of the environment, (2) code the degree of uncertainty between cue-outcome relationships, and (3) elicit molecular cascades strengthening or weakening the re-activated network. Consequently, any novel cue-outcome association can either be integrated in an existing memory or new memories can be laid down instead.

## Conclusion

In this review, we have tried to illustrate: first, how dopamine release is not only critical for learning but also to motivate animals to learn about the world at times of uncertainty; second, how past experience can shape dopamine-dependent learning; and, third, how dopamine might play a role not only in the initial learning of cue-reward associations but also in determining when to use stored experience and also when to consolidate associations between stimuli and outcome into memory. Furthermore, we have gone on to suggest ways in which the hippocampus might interact with NAc dopamine to facilitate these processes and to enable animals to react to what is behaviorally relevant in the given environment.

As is common with most comparable reviews, we gladly acknowledge that there are still many details that remain to be fleshed out and many complexities that have been glossed over for the sake of coherence. For instance, throughout, we have concentrated mainly on phasic dopamine release at the expense of slower tonic changes (although the two are likely related). As was discussed in an earlier section, even at the “slow, phasic” timescale (~0.5–10 s post-event) dopamine activity can evolve over time to represent several different parameters. Similarly, quite how different regions of the hippocampus interact with dopamine transmission across different parts of the striatum during learning and behavior remains to be determined. We would contend that a general computation might be shared across structures (for instance, determining statistical regularities of events and inhibiting ongoing behavior when there is conflict, for the hippocampus), even if the specific information provided by, for instance, ventral hippocampus to the NAc shell region may be different to dorsal hippocampus and NAc core.

Finally, while we have focused on hippocampus—VTA—NAc loops for simplicity, it is improbable that other temporal and frontal lobe regions are not also required to enable these processes to work optimally. For instance, orbitofrontal cortex, which receives hippocampal input and projects to the VTA, has been shown to provide input to allow dopamine cells to disambiguate similar states (for instance, being in a reward port following a choice), particularly when there is a delay between a choice and its consequences (Takahashi et al., 2011). Basolateral amygdala can also attenuate NAc cue-driven dopamine (Jones et al., 2010). How different nodes in this network interact to generate appropriate learning and behavior will be key questions to be addressed over the coming years in order to enable us to understand these processes in the complex, changeable and uncertain environments within which we live. Our hope is that, by investigating these networks more deeply and their interactions with dopamine release at different timescales, we may gain new insights to understand pathologies related to dopamine dysfunction such as schizophrenia where learning and behavior can become unconstrained by the parameters of the local environment.

### Conflict of interest statement

The authors declare that the research was conducted in the absence of any commercial or financial relationships that could be construed as a potential conflict of interest.
